# Comparative Study of Protection against Newcastle Disease in Young Broilers Administered Natural Chicken Alpha Interferon via Oral and Intramuscular Routes

**DOI:** 10.1128/mSphere.00585-20

**Published:** 2020-07-15

**Authors:** Faisal Rasheed Anjum, Sajjad Ur Rahman, Muhammad Aamir Aslam, Anas Sarwar Qureshi

**Affiliations:** a Institute of Microbiology, University of Agriculture, Faisalabad, Pakistan; b Department of Anatomy, Faculty of Veterinary Science, University of Agriculture, Faisalabad, Pakistan; University of North Carolina, Chapel Hill

**Keywords:** chicken IFN-α, Newcastle disease, therapeutic, innate immunity

## Abstract

Despite extensive vaccination approaches, Newcastle disease (ND) remains a permanent threat to the poultry industry worldwide. Besides vaccination, there is a burgeoning demand for new antivirals for use in interventions to control ND. One strategy is to strengthen the host innate immunity via host-derived innate immune proteins. Type I interferons define one of the first lines of innate immune defense against viral infections. Chicken interferon alpha (chIFN-α) is one of the potent cytokines that trigger antiviral responses. In the current study, we investigated the therapeutic effect of natural chIFN-α administered via oral and intramuscular (i.m.) routes against ND in broiler chickens. Our results showed that the level of protection against ND in response to chIFN-α therapy was dependent on the route and dose of IFN administration. A better therapeutic effect was observed in chickens treated with chIFN-α via the oral route than in those treated via the i.m. route. Regardless of the administration route, double-dose chIFN-α (2,000-U) treatments provided better protection than single-dose (1,000-U) treatments. However, complete protection against ND was achieved in birds treated with repeated doses of chIFN-α via the oral route. Histopathology of trachea, proventriculus, spleen, and liver showed a significant improvement in ND-induced degenerative changes in double-dose IFN-treatment groups compared to single-dose groups. Results of the hemagglutination test demonstrated a decrease in ND virus (NDV) titer in IFN-treated groups. Also, double doses of chIFN-α via oral route resulted in early recovery in weight gain. We propose that chIFN-α therapy via oral route could be an important therapeutic tool to control NDV infection in chicken.

**IMPORTANCE** Newcastle disease (ND) is an economically important contagious disease of wild and domestic birds worldwide. The disease causes severe economic losses in terms of production due to high mortality and morbidity in nonvaccinated chickens. Despite extensive vaccination approaches, Newcastle disease (ND) remains a permanent threat to the poultry industry worldwide. In the current study, we used natural chicken IFN-α as an innate immune modulator to counteract ND in chickens. We report that chIFN-α is effective in protecting the chickens against ND and also prevents shedding of the virus, which can then prevent further spread of the disease. We propose that in addition to vaccination, chIFN-α therapy could be an effective option for controlling ND in areas of endemicity.

## Abstract (precis)

Newcastle disease (ND) is an economically important contagious disease of wild and domestic birds worldwide. The disease causes severe economic losses in terms of production due to high mortality and morbidity in nonvaccinated chickens. Despite extensive vaccination approaches, Newcastle disease (ND) remains a permanent threat to the poultry industry worldwide. In the current study, we used natural chicken IFN-α as an innate immune modulator to counteract ND in chickens. We report that chIFN-α is effective in protecting the chickens against ND and also prevents shedding of the virus, which can then prevent further spread of the disease. We propose that in addition to vaccination, chIFN-α therapy could be an effective option for controlling ND in areas of endemicity.

## INTRODUCTION

Newcastle disease (ND) is an economically important contagious disease of wild and domestic birds worldwide and is caused by a negative-sense single-stranded RNA virus known as avian paramyxovirus serotype 1 (APMV-1) (1, 2). The disease is highly contagious and causes severe economic losses in terms of production due to high mortality and morbidity in nonvaccinated chickens. ND is also associated with reduced egg production in well-vaccinated layers (1). This disease is a constant threat to the poultry industry due to its continuous presence in the developing countries (3). Current strategies for the prevention of ND in developing countries include strict biosecurity measures and aggressive vaccination approaches (using live and killed vaccines) (1, 2). Live vaccines require a cold chain until the point of application to the birds, although this limitation has been overcome by the use of thermostable vaccine strains (4). Even then, live viruses may revert to a virulent form and cause clinical disease in the vaccinated birds. On the basis of the vaccine strain used for immunization, live vaccines may result in postvaccination respiratory reactions in younger birds which, in severe cases, may in turn result in a predisposition of the birds to secondary bacterial infections (2, 5). Also, the efficacy of such vaccines is reduced due to the presence of maternal antibodies (Abs) in the young birds (3). On the other hand, killed vaccines are less immunogenic due to the possible inactivation of certain immunogenic epitopes on the virus surface (6). Therefore, killed vaccines must be synchronized with live vaccine schedules and may require adjuvants to provoke a more pronounced immune response in birds (7). An undesirable reaction may also develop as a result of the presence of adjuvants in the vaccinated birds (8).

Neither biosecurity strategies nor vaccination strategies are sufficient to eradicate the circulating ND virus (NDV) strains, which remain endemic and may infect poorly vaccinated and unvaccinated birds (3, 9). Also, these conventional vaccines are unable to inhibit replication and shedding of other different NDV strains circulating in the poultry birds, which may serve as a source of continuous infection (2, 10). Although conventional vaccines remain in the mainstay to control ND in poultry, nevertheless, the hunt for better alternatives is still going on and has led to the emergence of novel agents, i.e., type I interferons (IFNs).

IFNs are the family of pleiotropic cytokines and are crucial for host innate immune defense against a variety of pathogens (11, 12). Generally, IFNs are classified into three main types: type I IFNs, type II IFNs, and type III IFNs (13). Chicken type I IFNs (chIFNs) represent the key constituent of the chicken innate immune system and protect the host from the invading viral pathogens. Three subtypes of chicken type I IFNs are chIFN-α, chIFN-β, and chIFN-κ (14, 15). Although the three members of the chicken type I IFN family are structurally diverse, they nevertheless bind to the same complex of heterodimer receptors composed of IFNAR1 and IFNAR2 chains (14, 16). Binding of type I IFNs to their corresponding receptor complex initiates a cascade of intracellular events, which ultimately results in the activation of several hundred IFN-stimulated genes (ISGs) via the JAK-STAT pathway (16, 17). Several studies have suggested the antiviral activity of recombinant chIFN-α against avian influenza virus (AIV), Newcastle disease virus (NDV), infectious bursal disease virus (IBDV), and infectious bronchitis virus (IBV) (18–20). However, the therapeutic effect of natural chIFN-α has not been demonstrated systemically. The current study produced the first comprehensive report that describes the comparative levels of efficacy of natural chIFN-α as a therapeutic agent against ND in a dose-dependent manner.

## RESULTS

### ND virus propagation and chIFN-α production.

**FIG 1:**
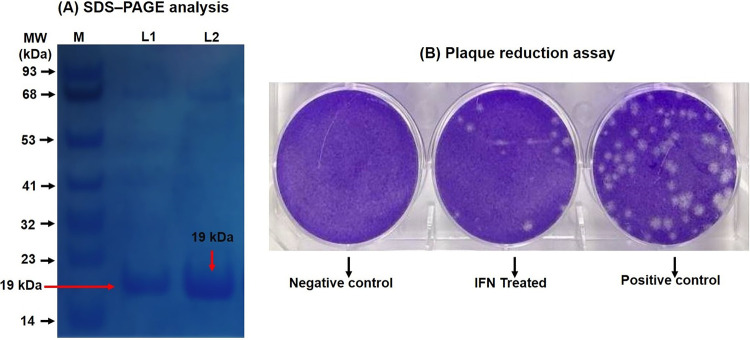
(A) SDS-PAGE analysis of the chIFN-α protein fractions obtained after various purification steps. Lane M is the protein ladder, while lane 1 (L1) and L2 represent the two similar bands (red arrows) of chIFN-α protein having a molecular weight of 19 kDa obtained after purification of CEF cell supernatant via controlled pore glass chromatography and cation-exchange chromatography and after treatment with anti-chIFN-β antibodies. (B) *In vitro* antiviral activity of purified chIFN-α determined via plaque reduction assay. Numbers of plaques were significantly reduced in IFN-treated (100 U/ml) CEF cells in comparison to the positive control, where no IFN treatment was provided.

After 48 h of NDV inoculation, allantoic fluid from embryonated chicken eggs was harvested and the hemagglutination titer was determined. A high NDV titer (1/526) was observed. The infectivity titer of the NDV in the harvested allantoic fluid was 10^5^ 50% egg infective doses (EID_50_)/0.1 ml. The mean embryo death time (MEDT) of 70 h and the intracerebral pathogenicity index (ICPI) value of 1.2 indicated the mesogenic nature of the Mukteshwar strain used in the current study. ChIFN-α in cell culture supernatant was purified in a two-step procedure that included pore glass chromatography and cation-exchange chromatography followed by final treatment of the eluent with anti-chIFN-β antibodies before analysis was performed using SDS-PAGE. Lane 1 (L1) and L2 in Fig. 1A represent the two similar protein bands of chIFN-α with a molecular weight (MW) of 19 kDa obtained via SDS-PAGE analysis of two samples collected from the same single purified chIFN-α product recovered after three purification steps, i.e., pore glass chromatography, cation-exchange chromatography, and treatment with anti-chIFN-β antibodies. These protein bands were eluted and further analyzed for their antiviral activity via plaque reduction assay (Fig. 1B). As is clearly visible in Fig. 1B, the addition of 100 U/ml of chIFN-α resulted in a significant reduction in the number of plaques produced by NDV in an IFN-treated well compared to the positive-control results (no-IFN treatment). Chicken IFN-α with an antiviral activity of approximately 1 × 10^7^ U/mg was recovered and reconstituted to a final concentration of 1,000 U/0.5 ml for use in the subsequent experiments.

### Results of *in vivo* studies of chIFN-α administered via oral route (experiment 1).

**FIG 2:**
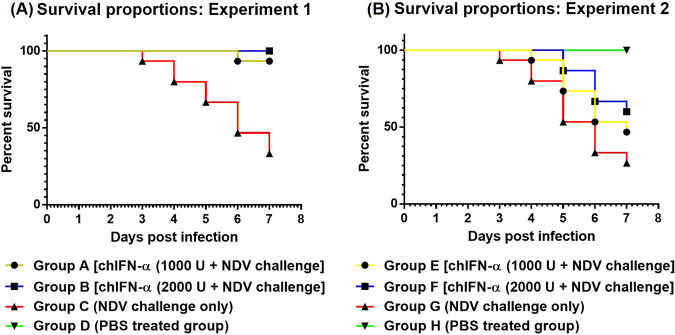
(A) Percent survival proportion of birds of experiment 1 administered chIFN-α via oral route. (B) Percent survival proportion of birds of experiment 2 treated with chIFN-α via intramuscular route.

Panel A of Fig. 2 highlights the percent survival proportion of all groups of experiment 1 administered chIFN-α via oral route. Morbidity rates among all four groups (A, B, C, and D) were determined based on the severity of clinical signs and symptoms. High rates of morbidity (80%) and mortality (66.7%) were observed in the positive-control group (group C), while no morbidity or mortality was observed in group D birds (negative-control group). Only a 6.7% mortality rate was observed in group A birds administered a single dose (1,000 U) of chIFN-α. On the other hand, no mortality was observed in group B birds treated with a double dose (2,000 U) of chIFN-α. Although the group A birds showed a lower mortality rate, only mild clinical signs and symptoms (mild respiratory signs) were observed in the group A birds. No such clinical signs and symptoms were observed in group B birds.

### Results of *in vivo* studies of chIFN-α administered via intramuscular route (experiment 2).

Panel B of Fig. 2 presents the proportion of birds that survived in all groups of experiment 2. In comparison to the results of experiment 1, a different pattern was observed in the mortality and morbidity rates of both IFN-treated groups (groups E and F) treated via the intramuscular (i.m.) route. The birds of group E administered single doses of chIFN-α (1,000 U) showed a relatively higher mortality rate (53.3%) than the birds in the double-dose group F (40%). However, this difference in percent morality of group E and F in response to administration of single and double doses of IFN was statistically nonsignificant (*P* > 0.05) as determined by Fisher's exact test. The results showing the morbidity and mortality rates in group G (positive-control group) and group H (negative-control group) of experiment 2 were rather similar to those seen with group C and D birds of experiment 1, respectively.

### Results of histopathological examination.

**FIG 3:**
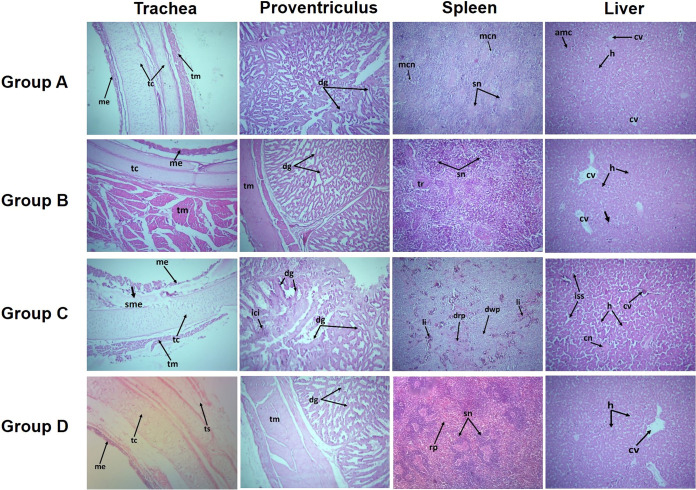
Histological micrograph of different organs of chickens collected from the different groups of experiment 1 (H&E [magnification, ×100]). Group A, chIFN-α (1,000 U) plus NDV; group B, chIFN-α (2,000 U) plus NDV; group C, NDV challenge only (positive-control group); group D, PBS treatment only (negative-control group). (Column 1) Trachea of group D showed normal mucosal epithelium with cilia and normal structure of the tracheal cartilage, tunica serosa with tracheal muscles. Trachea exhibited severe mucosal sloughing of mucosal epithelium and submucosal edema along with the infiltration of inflammatory cells in group C, while group B showed no degenerative changes in the epithelium, cartilage and tracheal muscles. Mild disruption in the mucosal epithelium with normal cartilage and tracheal muscles was observed in group A. (Column 2) Microscopically, the structure of proventriculus epithelium, glands, and muscles appeared normal in the negative-control group (group D) whereas group C showed severe desquamation of proventriculus glands and epithelium along with the infiltration of inflammatory cells into the submucosa of the proventriculus. Histology of group B tissue sections showed the normal structure of proventriculus. Mild desquamation of proventriculus glands was observed in group A. (Column 3) Histological examination showed that the normal splenic structure had a splenic nodule and red pulp in group D. NDV caused severe coagulative necrosis and lymphocyte infiltration in the splenic parenchyma in group C (positive-control group), and a disrupted appearance of the red and white pulp of splenic sections was observed in the members of that group. Splenic sections of group B exhibited an almost normal parenchymal structure. Group A splenic sections showed mild lymphoid depletion of white pulp and mild caseous necrosis. (Column 4) Normal hepatic structure was seen in the members of group D, with the central vein having a radiating arrangement of hepatocytes. NDV infection in group C induced severe necrosis surrounded by connective tissue, increased sinusoidal spaces, and hemorrhages in the hepatic tissues. Treatment with chIFN-α reversed these NDV-associated degenerative changes in hepatic sections of group B. On the other hand, mild hemorrhages and slight aggregation of mononuclear cells were witnessed in group A. Abbreviations: cn, caseous necrosis; cv, central vein; dg, degenerative glands; drp, disrupted red pulp; dwp, disrupted white pulp; h, hepatic cord; iss, increased sinusoidal spaces; ici, inflammatory cell infiltration; li, lymphocytic infiltration; mcn, mild caseous necrosis; me, mucosal epithelium; rp, red pulp; sme, sloughing of mucosal epithelium; sn, splenic nodule; tr, trabuclar artery; tc, tracheal cartilage; tm, tracheal muscles; ts, tunica serosa.

**FIG 4:**
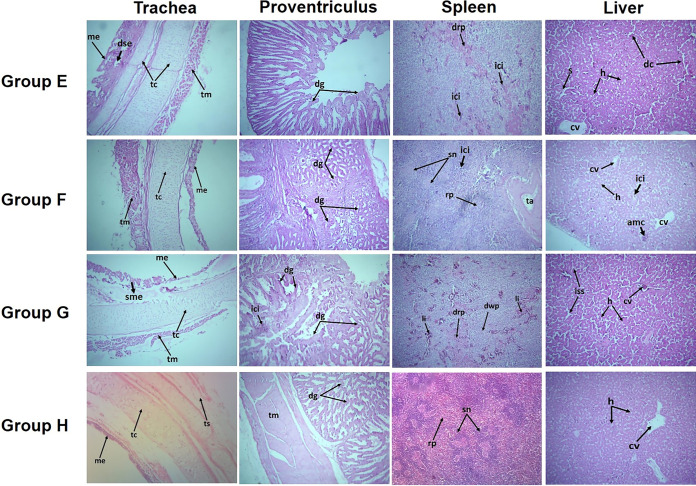
Histological micrograph of different organs of chickens collected from the different groups of experiment 2 (H&E [magnification, ×100]). Group E, chIFN-α (1,000 U) plus NDV; group F, chIFN-α (2,000 U) plus NDV; group G, NDV challenge only (positive-control group); group H, PBS-treated group (negative-control group). Note that all of the histopathological changes observed in the positive-control group (group G) and the negative-control group (group H) in experiment 2 were similar to those seen with group C (positive-control group) and group D (negative-control group) in experiment 1. (Column 1) Group E showed moderate degenerative changes in the tracheal epithelium and submucosal edema. Mild sloughing in the mucosal epithelium with normal cartilage and tracheal muscles was observed in group F. (Column 2) IFN treatment via the intramuscular route in group E resulted in a mild improvisation in NDV-induced degenerative changes in the histological structure of the proventriculus. The level of NDV-associated histopathological changes in proventriculus sections of group F was mild, representing an enhanced therapeutic effect of chIFN-α against NDV infection. (Column 3) Group F showed mild changes in the normal splenic parenchyma and infiltration of inflammatory cells. Tissue sections from the members of of group E exhibited infiltration of the inflammatory cells along with disturbed red and white pulp. (Column 4) NDV-induced degenerative changes in the liver sections of the members of group E included an asymmetrical arrangement of hepatic cords and sinusoidal cords around the central vein. Mild degenerative changes and aggregation of mononuclear cells were witnessed in group F. Abbreviations: amc, aggregation of mononuclear cells; cv, central vein; dse, degeneration of submucosal epithelium; dc, degenerative changes; dg, degenerative glands; drp, disrupted red pulp; dwp, disrupted white pulp; h, hepatic cord; iss, increased sinusoidal spaces; ici, infiltration of inflammatory cells; me, mucosal epithelium; rp, red pulp; s, sinusoidal cords; sme, sloughing of mucosal epithelium; sn, splenic nodule; tr, trabecular artery; tc, tracheal cartilage; tm, tracheal muscles; ts, tunica serosa.

Results of histopathological examination of the trachea, proventriculus, spleen, and liver specimens collected from the birds of experiments 1 and 2 are presented in Fig. 3 and 4, respectively. Severe degenerative changes, i.e., epithelial sloughing, hemorrhages, edema, and infiltration of lymphocytes, were observed in tissue sections collected from the birds of the positive-control groups of experiments 1 and 2. Histopathologically, group B birds showed no lesions in trachea, spleen, proventriculus, and liver. Although the group A chickens survived the severe form of the disease, their organs showed slight degenerative changes (less-severe lesions). Group C birds showed significant pathological changes in the trachea, proventriculus, spleen, and intestine (Fig. 3). Histopathological lesions observed in the positive-control group (group C) included hemorrhages in the tracheal lumen and lymphocyte infiltration, hemorrhages in proventriculus, disrupted intestinal glands, sloughing of the intestinal epithelium, and severe coagulative necrosis in the splenic parenchyma. Normal histological structures of all the organs were observed in group D (negative-control group).

Mild to moderate severity in pathological lesions was observed in both IFN-treated groups (group E and F) of experiment 2 (Fig. 4). Histopathological lesions observed in group E chickens included submucosal edema and disrupted tracheal epithelium, hemorrhages in proventriculus, sloughing of the intestinal epithelium, and infiltration of inflammatory cells in the splenic parenchyma. Histopathology of group F birds showed mild sloughing of the tracheal cartilage, petechial hemorrhages in proventriculus, and slight disruption in the epithelium of intestinal glands. No such lesions were observed in tissues of organs collected from the negative-control group (group H).

### ChIFN-α treatment resulted in reduced virus shedding.

**FIG 5:**
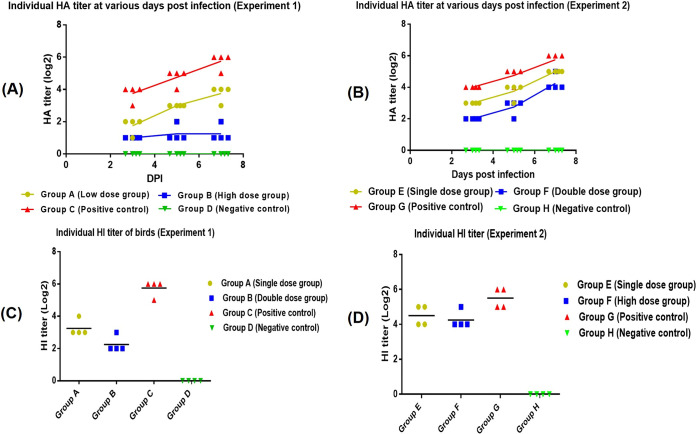
(A) Distribution of individual hemagglutination (HA) titers of the birds treated with chIFN-α via oral route. HA titers of all the groups (IFN treatment and control) in experiment 1 at various days postinfection are described. (B) Distribution of HA titers of birds administered chIFN-α via intramuscular route at various days postinfection. (C) Hemagglutination inhibition (HI) titers of the birds of experiment 1 at 7 days postinfection. Among all the birds in group A, evaluated for the presence of anti-NDV antibodies, three birds showed a log_2_ HI titer of 3 (1/8), while one bird showed a log_2_ HI titer of 4 (1/16). Similarly, one bird of group B resulted in a log_2_ HI titer of 3 (1/8), while the rest of the three birds exhibited a log_2_ HI titer of 2 (1/4). Among the four birds in group C, one bird showed a log_2_ HI titer of 5 (1/32), while the other three birds presented an HI titer of 6 (1/64). (D) HI titers of individual birds of experiment 2 at 7 days postinfection. A log_2_ HI titer of 4 (1/16) was observed for two birds of group E, while the other two birds showed a higher log_2_ HI titer of 5 (1/32) in group E. Three birds in group F showed a log_2_ HI titer of 4 (1/16), while one bird exhibited a log_2_ HI titer of 5 (1/32). Two birds in the positive-control group exhibited a log_2_ HI titer of 5 (1/32), while the other two showed a log_2_ HI titer of 6 (1/64). No antibodies were detected in group H (negative-control group).

Panel A of Fig. 5 presents the individual NDV titers of four groups of experiment 1 obtained by reisolation of virus in 9-day-old chicken embryonated eggs. Both the group A birds and group B birds showed a decrease in hemagglutinin (HA) titer at 3, 5, and 7 days postinfection (dpi) compared to the birds in group C (positive-control group), whereas no HA titer was observed in the negative-control group (group D). Group B birds treated with high doses of chIFN-α (2,000 U), in contrast to the birds in the positive-control group, showed a significant decrease in virus shedding throughout the experiment, although a lower dose of chIFN-α (1,000 U) reduced the NDV shedding for the first few days but failed to limit the NDV replication in the longer run (Fig. 5A).

The HA titers of individual birds administered chIFN-α via i.m. route are presented in Fig. 5B. Both low and high concentrations of chIFN-α reduced the virus shedding at 3, 5, and 7 dpi compared to the HA titers of group G birds (positive-control group) at the corresponding days. Although the high chIFN-α concentration was more effective in limiting NDV titer, this reduction in virus titer was observed for the first few days after IFN treatment. Regardless of chIFN-α concentration, a gradual increase in NDV titer was observed at 5 and 7 dpi for both IFN treatment groups (the low-dose and high-dose groups). However, the HA titer in both groups (groups E and F) remained lower than that seen with the positive-control group throughout the experiment.

### Determination of titers of anti-NDV antibodies.

Individual antibody titers (hemagglutination inhibition [HI] titer) of birds administered chIFN via the oral (experiment 1) and i.m. (experiment 2) route observed at 7 dpi are presented in Fig. 5C and D. In comparison to the positive-control group (group C), a decrease in HI titer was measured in birds administered chIFN-α via oral route (Fig. 5C). Among the two chIFN-treatment groups of experiment 1, group A birds reflected a higher HI titer than the group B birds at 7 dpi. No antibody titers were observed in group D birds treated with phosphate-buffered saline (PBS) only. A similar pattern in HI titers was also observed in all groups (groups E, F, G, and H) of experiment 2. Both groups E and F exhibited lower antihemagglutinin titers than the positive-control group (group G). Overall, chIFN-α administration via oral route (groups A and B) resulted in a lower humoral response than was seen with the corresponding groups treated with chIFN via i.m. route (groups E and F), in which a higher humoral immune response was observed.

### Effect of chIFN-α on body weight of chickens.

**FIG 6:**
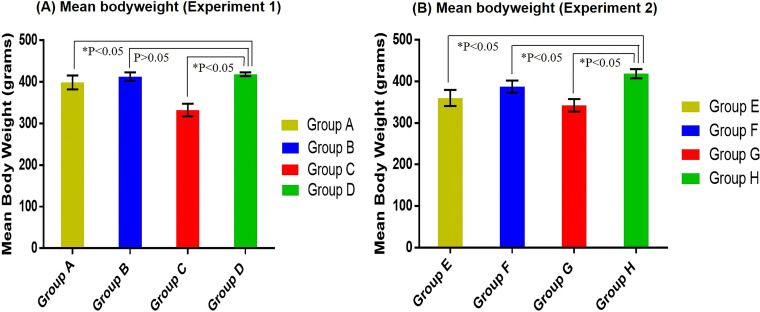
Comparison of mean body weights (in grams) of all the IFN-treated and untreated groups of experiment 1 (A) and experiment 2 (B). (A) In experiment 1, significantly lower (*P* < 0.05) mean body weight was observed in single-dose treatment group A and in the positive-control group (group C) than in the negative-control group (group D). The difference in the mean body weights of the double-dose group and the negative-control group was nonsignificant (*P* > 0.05). (B) In experiment 2, mean body weights of all three treated groups, i.e., groups E, F, and G, were significantly lower (*P* < 0.05) than those of the members of negative-control group H.

The mean body weights (in grams) of all groups (treatment and control) of experiments 1 and 2 are shown in Fig. 6A and B, respectively. In experiment 1, the four groups showed differences in mean body weights at 7 dpi. The mean body weights and standard deviations determined for all four groups in experiment 1 were as follows: for group A, 399 ± 16.6; for group B, 412.9 ± 10.4; for group C, 332.42 ± 15.1; for group D, 418.5 ± 4.7. Significantly lower mean body weights were observed in the positive-control group (group C) and in single-dose IFN treatment group A than in the negative-control group (group D). Although the group B birds showed a slightly lower mean body weight than the group D birds, the differences were not statistically significant (Fig. 6A). The mean body weight of the double-dose IFN treatment group was close to that of the negative-control group. This difference in mean body weights among the groups was due to NDV infection in birds. A high dose of chIFN-α resulted in better protection and, ultimately, earlier recovery in body weight gain than were seen with the other groups, i.e., groups A and C.

On the other hand, a different pattern in mean body weights of birds was observed in the chIFN-treated groups (groups E and F) and control groups (G and H) of experiment 2 (Fig. 6B). Significant differences in mean body weights were observed in group E, F, and G birds at 7 dpi compared with group H (negative-control group). The low-dose and high-dose chIFN-treatment groups (E and F) showed mean body weights of 360.3 g ± 19.4 g and 387.7 g ± 14.5 g, respectively, compared to the negative-control group (418.9 g ± 11.03 g). Similarly to the positive-control group of experiment A, a significantly lower mean body weight (342.7 g ± 15.1 g) was exhibited by the group G birds (positive-control group) of experiment 2.

## DISCUSSION

Currently, vaccination is the primary approach for controlling ND worldwide. However, due to many contributing factors, several ND outbreaks are reported across the globe. One strategy to control ND in poultry is to modify the host innate immune system by using type I IFNs. In the current study, we determined the therapeutic potential of natural chIFN-α against ND infection. Briefly, chIFN-α was produced in chicken embryo fibroblast (CEF) cells and used as an exogenous cytokine to strengthen the innate immune responses in young broiler chicks challenged with ND virus. We have used chIFN-α as a therapeutic agent against NDV. Although pretreatment of chIFN-α before virus challenge also provides a measured level of protection against diseases (21–24), previous findings of Meng et al. suggest that using chIFN-α as a therapeutic agent provides better protection against disease in comparison to the prophylactic use (19). In order to determine the optimum therapeutic activity of natural chIFN-α against NDV, two routes of administration were adopted, i.e., the oral and intramuscular routes. The results of our challenge protection studies suggested that chIFN-α administration via oral route is more effective than administration via i.m. route. Previous *in vivo* studies in chickens also reported that administration of chIFN-α via oral route inhibited H9N2 avian influenza virus (19). Marcus et al. also previously reported significant protection against ND in young chickens administered chIFN-α via oral route (24). Protective effects of chIFN-α administered via oral route against infectious bronchitis in chickens have also been demonstrated previously (18). In our study, a repeated dose of chIFN-α resulted in enhanced protection against ND infection via oral route compared to the single-dose therapy via the same route. A slightly higher mortality rate (6.7%) was observed in single-dose IFN-treated birds (group A) than in double-dose group B birds administered IFN via oral route. The group A birds showed a lower mortality rate, and only mild clinical signs and symptoms (respiratory signs and viremia) were observed in most of the group A birds. No such clinical signs and symptoms were observed in double-dose IFN treatment group B. ChIFN-α treatment via i.m. route did not provide adequate protection to the birds against NDV infection, as high mortality rates of 53.3% and 40% were observed in single-dose treatment group E and double-dose group F, respectively. These differences in the performance of chIFN-α therapy administered via oral and i.m. routes might be due to many factors such as route of administration, timing of IFN therapy, and the dose of chIFN-α (20, 25). There is a possibility that chIFN-α administration via i.m. route might have restricted the activity of NDV in circulation but not on the mucosal surfaces. With the passage of time, a decline in serum level of chIFN-α might allow the residual ND virus to replicate and infect the birds, resulting in a higher mortality rate (20). Our results support this hypothesis, as repeated IFN therapy via i.m. route (group F) may have resulted in longer bioavailability of chIFN-α in serum over the course of experiment and consequently in better protection from the infection compared to single-dose treatment (group E). The timing of IFN administration can also affect the degree of disease outcome. Previous studies have suggested that IFN administration before the appearance of clinical signs has resulted in significant protection against the diseases (19, 20). Also, the optimum dose of IFN required for complete protection against a disease might be associated with a number of factors, i.e., age of the birds, pathogenicity of the virus, and health status of the birds at the time of the administration. Histopathological examination of various organs of IFN-treated and untreated birds infected with NDV showed that chIFN-α therapy ameliorated the pathology of Newcastle disease. Double doses of chIFN-α administered via oral route significantly ameliorated the ND-induced degenerative changes in the trachea, liver, spleen, and proventriculus compared to the rest of the IFN-treated groups. Marcus et al. also previously described an amelioration of the pathology of the trachea in response to chIFN-α treatment via oral route (24).

The current study also demonstrated that chIFN-α therapy decreased the virus shedding by limiting the NDV replication, determined by detection of decreased HA titers in IFN-treatment groups compared to the positive controls. Previously, Jiang et al. also reported a decrease in AIV titer in response to chIFN treatment (22). The therapeutic effect of chIFN-α on the humoral immune response of chickens against NDV was also investigated in the current study. At 7 dpi, a decline in the antibody titers of birds administered chIFN-α via oral and i.m. routes was measured compared to the corresponding positive controls (groups C and G). It was observed that double doses of chIFN-α resulted in a decreased humoral response in comparison to single doses in both experiments 1 and 2. Overall, in both experiments, the level of anti-NDV antibodies was much lower in birds administered chIFN via oral route than in those administered chIFN via i.m. route. These results reflecting humoral immune responses were in accordance with the previous study conducted by Marcus et al. in which a somewhat similar pattern in HI titers was observed in chIFN-α-treated groups (24). The reason for the lower humoral response in the double-dose IFN group might be that the repeated chIFN-α therapy restricted the replication of the virus to such an extent that the NDV load was below the threshold for antibody stimulation. The absence of viremia in double-dose group B birds strengthens our hypothesis that a high dose of chIFN-α inhibited the NDV replication and thus resulted in a decreased Ab titer rather than in immune suppression. Further confirmation of our hypothesis was provided by the results of histopathology analysis of various organs in which a high dose of chIFN-α (group B) completely improved the NDV-induced pathological changes. A comparison of mean body weights of all the groups at 7 dpi indicated that double-dose chIFN-α treatment via oral route leads to a rapid recovery in weight gain compared to the other treatment groups. A similar trend in weight gain was also previously reported by Marcus et al. in which substantial differences were observed in the individual body weights of the IFN-treated group and the virus-challenged positive-control group (24). Our findings were not in accordance with the study conducted by Meng et al. in which no significant differences were determined in body weight gain of virus-challenged and IFN-treated young birds (7 days old). However, the trend in body weight gain reported for 33-day-old birds (19) was in accordance with the results of our study. Here, we report that chIFN-α is effective in protecting the chickens against ND and also prevents shedding of the virus, which can then prevent further spread of the disease. We propose that in addition to vaccination, chIFN-α therapy could be an effective option for controlling ND in areas of endemicity. We speculate that these results could be extended to treat other viral diseases in poultry.

## MATERIALS AND METHODS

### Birds.

A total of 120 (1-day-old) grade A broiler chicks were purchased from the local hatchery in Faisalabad, Pakistan. All the birds were kept under good management conditions. Birds were provided with feed and water *ad libitum* with a standard broiler starter and grower diet plan throughout the experiment. At day 7, birds were tested for the presence of maternal anti-NDV antibodies via hemagglutination inhibition (HI) testing. The present study was conducted in accordance with the rules and regulations of the Institutional Bioethics Committee (IBC), University of Agriculture, Faisalabad, Pakistan.

### Virus and cell culture.

Newcastle disease virus (Mukteshwar strain) was kindly provided by Veterinary Research Institute (VRI), Lahore, Pakistan. NDV was further propagated in the allantoic cavity of 9-day-old specific-pathogen-free (SPF) chicken embryonated eggs according to the standard procedures as described previously (26). Briefly, NDV was inoculated in the allantoic fluid and incubated for 72 h at 37°C. The embryos that died within the first 24 h were discarded. After 72 h, the eggs were chilled overnight at 4°C followed by harvesting of the allantoic fluid. The harvested allantoic fluid was first concentrated by low-speed centrifugation at 4°C, and the supernatant was aspirated. Supernatant was supplemented with gentamicin (30 μg/ml) and stored at –20°C until use. Hemagglutination (HA) testing was performed to determine the titer of ND virus. Further confirmation regarding the pathotype of the Mukteshwar strain was done by the use of two *in vivo* tests, including determination of the mean embryo death time (MEDT) in chicken embryonated eggs and determination of the intracerebral pathogenicity index (ICPI) in 1-day-old chickens, according to the standard protocol as described previously (27, 28). The EID_50_ of the virus was calculated by the method of Reed and Muench as described previously (29).

Chicken embryo fibroblast (CEF) cells were derived from SPF chicken embryos (9 days old) and cultured in complete Eagle’s minimal essential medium (Gibco, USA) supplemented with fetal bovine serum (10%), 1× gentamicin sulfate, tryptose phosphate broth (5%), and l-glutamine (0.02%) according to the standard procedure as described previously (30).

### ChIFN-α production and quantification.

Chicken IFN-α was produced in CEF cells as described previously (31). Briefly, CEF cells were seeded in 50-cm^3^ cell culture flasks (1 × 10^7^ cells per flask) and incubated at 38°C in a humidified (80%) incubator under 5% CO_2_ for 48 h. After a confluent monolayer was formed, each cell culture flask was infected with 0.1 ml of NDV (10^8^ PFU). After adsorption for 1 h at 38°C, virus inoculum was removed, and fresh medium was added after washing of the cell sheet. After 24 h of stimulation with NDV, the cell culture supernatants were harvested and centrifuged at 500 × *g* for 15 min to remove cellular debris. A second centrifugation (99,000 × *g*) was performed for 2 h to eliminate the excess virus followed by heating at 65°C to inactivate any residual virus, and the reaction mixture was stored at –20°C until use. A chicken IFN-α enzyme-linked immunosorbent assay (ELISA) kit (MyBioSource, Inc., USA) was used to detect the chIFN-α in the supernatant. Purification of chIFN-α from cell culture supernatant was carried out via pore glass chromatography and cation-exchange chromatography. To remove the chIFN-β, fractions containing the concentrated protein were treated with anti-chIFN-β antibodies (Bio-Rad, USA) followed by analysis using SDS-PAGE containing a polyacrylamide gel (15%) and were stained with Coomassie blue. Biological activity of chIFN-α was determined via IFN bioassay according to previously described protocols (32).

### Determination of the therapeutic potential of natural chIFN-α.

**FIG 7:**
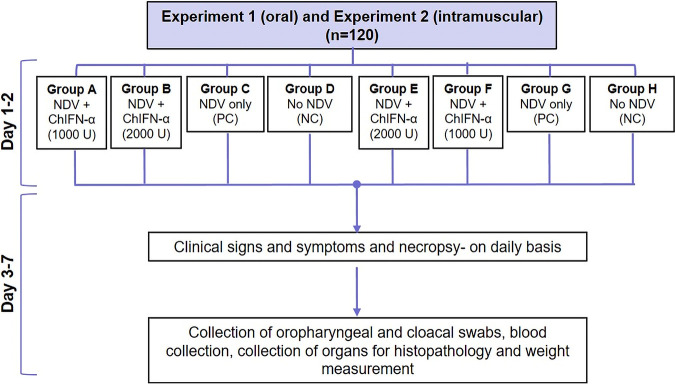
Experimental layout for the determination of the therapeutic potential of natural chIFN-α against NDV via the oral route (experiment 1) and the intramuscular route (experiment 2). The birds (*n* = 120) were equally divided into 8 groups (designated A to H), with each group composed of 15 birds (*n* = 15). Experiment 1 included the first four groups (A to D), while experiment 2 comprised groups E, F, G, and H. PC, positive control; NC, negative control.

To determine the therapeutic effect of natural chIFN-α against ND virus, two experiments were conducted (Fig. 7).

**(i) Experiment 1.** A total of 60 (7-day-old) broiler chicks were divided into four groups (groups A, B, C, and D), with each group comprising 15 birds (Fig. 7). Birds of groups A, B, and C were challenged with the ND virus (10^5^ EID_50_/0.1 ml) via intraocular drops. Each bird in groups A and B was administered 0.5 ml of a solution containing 1,000 U of chIFN-α via oral route, 8 h after the virus challenge. Group B birds were again administered 1,000 U of chIFN-α, 12 h after the first dose (Fig. 7). Group C birds were kept as the positive-control group. Group D birds (negative-control group) were inoculated with PBS only. To determine the therapeutic effect of chIFN-α against ND, clinical signs, viremia, pathological changes, and percent survival rate were determined until 7 days post-NDV challenge.

**(ii) Experiment 2.** Sixty broiler chicks (7 days old) were evenly divided into four groups (groups E, F, G, and H). Each bird in groups E, F, and G was challenged with NDV (10^5^ EID_50_/0.1 ml) via intraocular route. After 8 h of NDV challenge, all the birds in group E and group F were administered a 0.5-ml solution containing 1,000 U of chIFN-α via intramuscular (i.m.) route. A second dose of chIFN-α (1,000 U) was repeated in group F birds, 12 h after the first dose (Fig. 7). The negative-control group (group H) was inoculated with PBS only, while group G was maintained as the positive-control group. Clinical signs, viremia, pathological changes, and the survival rate were recorded until 7 days post-NDV challenge.

**(iii) Histopathological examination.** To determine the protective effect of chIFN-α against NDV infection, histopathological examinations were performed. For this purpose, various organs, i.e., trachea, liver, spleen, and proventriculus, were collected from birds of all the groups of experiments 1 and 2 at the end of each experiment. All the tissue samples were first fixed in neutral buffer formalin solution (10%) followed by processing via paraffin embedding technique as described previously (33). Tissue blocks were cut into 5-μm thicknesses and mounted on frosted glass slides followed by staining of tissue sections with hematoxylin and eosin (H&E) stains. To determine any cellular degenerative changes in all IFN-treated and untreated groups, stained tissue sections were examined under a microscope (magnification, ×100). Assessment of histopathological lesions in response to NDV infection was done descriptively, and the severity level (mild, moderate, or severe) of degenerative changes was determined on the basis of the distribution of focal lesions, presence of degeneration, edema, hemorrhages, necrosis, and infiltration of inflammatory cells.

**(iv) Determination of virus shedding via hemagglutination test.** Whether the chIFN-α treatment had resulted in reduced virus shedding or not, oropharyngeal and cloacal swabs were collected from birds at 3, 5, and 7 days after virus challenge. Swab samples were inoculated in chicken embryonated eggs (9 days old) followed by incubation at 38°C. Hemagglutination assay (HA) (34) was used to characterize the NDV titer in all treatment and control groups of both experiments 1 and 2.

**(v) Determination of antibody titer via hemagglutination inhibition test.** To determine the titer of antibodies produced in response to NDV hemagglutinin, blood samples were collected at 7 dpi (days postinfection) from all IFN-treated and untreated groups of experiments 1 and 2 followed by separation of the serum. Standard hemagglutination inhibition (HI) testing was used to determine the presence of anti-NDV antibodies in the serum samples (34).

**(vi) Effect of chIFN-α on growth performance of chickens.** Body weight gain was measured at the end of the experiment to determine any significant differences among the IFN-treated and untreated groups of both experiments. Comparisons of chIFN-treated and control groups were performed using Student's *t* test. Multiple comparisons of treatment groups were analyzed using one-way analysis of variance (ANOVA).
